# Feeling Touched: Emotional Modulation of Somatosensory Potentials to Interpersonal Touch

**DOI:** 10.1038/srep40504

**Published:** 2017-01-12

**Authors:** N. Ravaja, V. Harjunen, I. Ahmed, G. Jacucci, M. M. Spapé

**Affiliations:** 1 Helsinki Institute for Information Technology HIIT, Department of Computer Science, Aalto University, Espoo, Finland; 2 Helsinki Collegium for Advanced Studies, University of Helsinki, Helsinki, Finland; 3 School of Business, Aalto University, Helsinki, Finland; 4 Department of Social Research, University of Helsinki, Helsinki, Finland; 5 Helsinki Institute for Information Technology HIIT, Department of Computer Science, University of Helsinki, Helsinki, Finland.

## Abstract

Although the previous studies have shown that an emotional context may alter touch processing, it is not clear how visual contextual information modulates the sensory signals, and at what levels does this modulation take place. Therefore, we investigated how a toucher’s emotional expressions (anger, happiness, fear, and sadness) modulate touchee’s somatosensory-evoked potentials (SEPs) in different temporal ranges. Participants were presented with tactile stimulation appearing to originate from expressive characters in virtual reality. Touch processing was indexed using SEPs, and self-reports of touch experience were collected. Early potentials were found to be amplified after angry, happy and sad facial expressions, while late potentials were amplified after anger but attenuated after happiness. These effects were related to two stages of emotional modulation of tactile perception: anticipation and interpretation. The findings show that not only does touch affect emotion, but also emotional expressions affect touch perception. The affective modulation of touch was initially obtained as early as 25 ms after the touch onset suggesting that emotional context is integrated to the tactile sensation at a very early stage.

Human touch has been suggested to evoke a sense of “proximity and establish the human connection”^1^. Our sense of touch has been shown to play a large role in establishing how we feel and act toward another person. Hostility, nurturance, dependence, and affiliation are some of the social messages that can be conveyed through touch^2^. The effect that people are more altruistic, in the sense that touch can elicit measurable generosity (e.g., waiters who touch their customers get larger tips), has been termed the Golden, or Midas, touch (after the mythological king^3^). The effects of interpersonal touch (a cue to friendship) by an outgroup member have also been found to extend beyond the toucher to the toucher’s social group, thereby reducing (implicit) ethnic prejudice^4^. It is evident, however, that the interpretation of touch can wildly vary as a function of the social context^5^. Accordingly, the present study was designed to examine how different facial emotional expressions modulate the processing of touch as indexed by somatosensory-evoked potentials (SEPs), the event-related potential (ERP) in EEG resulting from touch.

Touch and emotion are deeply connected. For example, in many languages, our words for tactile sensation (feeling) and emotional sensation (feeling) strongly overlap. Also relevant to the connection of touch with emotion, Ellingsen *et al*.^6^ found that, after an intranasal oxytocin treatment, a gentle human touch sharpened ratings of friendliness and attractiveness, so that images of frowning faces were rated as less friendly and attractive, whereas smiling faces were rated as more friendly and attractive. Likewise, Schirmer *et al*.^7^ showed that touch may sensitize ongoing emotional processes and that this sensitization is mediated by bottom-up somatosensory processing. They presented neutral and negative pictures preceded or not by a touch and found that the difference in the late positive component (LPC) of the ERP normally observed between emotional and neutral items was increased (i.e., enhanced emotion discrimination) by touch regardless of whether the touch was performed by a friend or a mechanical device.

However, not only does touch affect emotional processes, but the processing of touch may also be modulated by contextual factors. Touch does not universally lead to positive emotions. Cultural differences can result in touch being construed as a breach of preferred interpersonal distance^8^. The appreciation of touch also varies within a culture: Henley^9^ noticed that people with higher social status more frequently engaged in interpersonal touch and suggested that a touch can be a reminder of an inferior social position. Furthermore, a touch from a woman has been shown to elicit positive Midas Touch effects for both genders^10^, while a “male touch”, in some cultures, can even result in inverse effects^11^. Likewise, we recently showed that the message implied by the touch and the relationship between sender and receiver both are critical: a touch along with a friend’s unfair request negatively affected generosity^12^. Gazzola *et al*.^13^ also showed that heterosexual male subjects who received a “sensual touch” in the MRI scanner showed different patterns of activity in their somatosensory cortex if they thought the source of the touch was a male rather than a female, even though the touch was always administered by a female. Evidently, emotional expressions are one important external contextual factor that may affect basic tactile perception. Recently, Ellingsen *et al*.^6^ found that (static images of) smiling faces increased, whereas frowning faces reduced, pleasantness of concomitant touch. Therefore, we expected that a touch preceded by a toucher’s angry facial expression will be rated as less pleasant and more intense compared to other facial expressions.

Studying SEPs is particularly useful when investigating how tactile processing is modulated by emotional expression as the temporal resolution allows us to precisely understand *when* a toucher’s emotional expressions modulate tactile perception and thus at what stage of processing emotion and touch are related. For example, if different emotional expressions immediately cause differences in activity in the somatosensory cortex (SCx), it follows that emotional modulation happens before extraction of tactile features—as enabled by the SCx itself—is completed. Some recent studies indeed showed remarkably early effects of emotion on SEPs: viewing static emotional pictures (images of facial expressions or with other emotional content) was found to modulate SEPs to touch delivered to the hand^14^ and/or face^15^. We^12^ similarly showed that emotional stimuli—fair and unfair propositions—modulated touch processing but found the effect in the later (>200 ms) temporal range of the SEP and in the reverse direction. In general, however, it is not clear how visual contextual information modulates the sensory signals, and at what levels does this modulation take place^5^. Therefore, in the present study, we asked how a toucher’s emotional expressions (anger, happiness, fear, and sadness) modulate touchee’s SEPs in different temporal ranges.

Although the previous studies show that an emotional context may alter how touch is processed, they have used static emotional images that cannot create an impression that both the touch and emotional expression emanate from the same actor. Obviously, the common social touch is rarely experienced in a disembodied manner, occurring instead within an interpersonal context in which touch and emotional expressions are interlinked. However, a direct investigation of how emotional expressions modulate a truly *interpersonal* touch has thus far remained a technical impossibility. This would require seeing the emotional facial expressions of others and feeling their touch, while precisely controlling both. Emotional expressions are also unlike pictures of facial affect^16^ in that they are dynamic. The temporal parameters of expressions should be known and controlled to avoid confounds. The physical parameters of a touch should be constant, with its timing of delivery accurate to the millisecond. Finally, the noise inherent in EEG means this degree of control should be repeated hundreds of times.

In the present study, we fulfil these requirements and show how a facial expression affects experience of, and cortical response to, the touch of our interaction partner (virtual character). The experiment uses virtual reality, hand tracking and tactile gloves (see Fig. 1) to create the impression (see Fig. 2) that another person expresses an emotion and then reaches out to touch the participant’s hand. Unlike the previous studies, we investigated multiple basic emotional expressions to better align with multidimensional theories of emotion^17^.

## Results

Emotion recognition was high on average, with 86.3% ± 1.5% correct classification. EEG data of two participants and behavioural data of one participant were lost due to technical problems and removed from the analyses.

### Behaviour

To understand how people perceived both touch and emotional expression, we analysed their subjective ratings. Ratings of *intensity* and *forcefulness* were combined (r = 0.68), as were ratings of *pleasantness* and *friendliness* (r = 0.48). This resulted in four measures (*intensity* and *pleasantness of touch*, and of *expression*) that were analysed using four repeated-measures analyses of variance (ANOVAs) with *touch type* (vibration, mechanical) and *emotional expression* (neutral, anger, happiness, fear and sadness) as factors. Given the four separate ANOVAs, an alpha of 0.0125 (0.05/4) was used to control for type I errors.

*Perceived intensity of expression* depended on *emotional expression*, F (3.14, 125.64) = 43.79, η^2^ = 0.52, MSE = 0.93, p < 0.0001, but not on *touch type*, p = 0.14. As might be imagined, an angry expression was rated as more intense than any other expression (*p*s < 0.0001), and happy and fearful expressions were rated as more intense than sad and neutral expressions (*p*s < 0.0001). No differences were found between the happy and fearful (*p* = 0.16) or between the sad and neutral condition (*p* = 0.73).

*Pleasantness of expression* was also affected by *emotional expression*, F (2.58, 103.32) = 47.78, η^2^ = 0.54, MSE = 1.35, p < 0.0001, but here, *touch type* also had a significant main effect, F (1, 40) = 8.93, MSE = 0.70, η^2^ = 0.13, p = 0.005. Post hoc tests revealed that all expressions differed significantly from each other (*p*s < 0.033). Angry expressions were rated as least, while happy expressions were rated as most, pleasant, fearful, sad, and neutral being in the between. Expressions preceding a mechanical touch were rated as more pleasant than those preceding the vibration touch.

*Intensity of touch* was significantly affected by *emotional expression*, F (2.05, 81.79) = 16.30, MSE = 0.65, η^2^ = 0.29, p < 0.0001, but not by *touch type*, p = 0.05. A touch preceded by an angry expression was perceived as more intense than any other touch (*p*s < 0.0001) while happy and fearful touch was perceived as more intense than sad and neutral touch (*p*s < 0.018).

Finally, *pleasantness of touch* was found to depend on both the type of touch, F (1, 40) = 60.87, MSE = 1.78, η^2^ = 0.60, p < 0.0001, and *emotional expression,* F (2.72, 108.89) = 60.86, MSE = 1.78, η^2^ = 0.60, p < 0.0001. Mechanical touch was rated as more pleasant (1.03 ± 0.13 points) than vibration touch. Touches preceded by a happy or sad expression were rated as most pleasant while angry-, fearful-, and neutral-expression touches were perceived as less pleasant (*p*s < 0.045). No difference was found between happy- and sad- (*p* = 0.28) or between happy- and fearful-expression (*p* = 0.62) touch.

Thus, as can be observed from Fig. 3, *emotional expression* affected touch perception: the same touch was perceived differently depending on the expressed emotion. This was true for both affective ratings (“how pleasant was the touch?”), and for basic, psychophysical judgments (“how intense was the touch?”).

### Early SEPs

Initial inspection of the grand averages revealed that the mechanical stimuli evoked imprecise early potentials. We suspected this to be due to random latency differences in the tactile glove, stemming from individual anatomic differences (e.g., in hand size and therefore stretched fabric), resulting in different haptic experiences. Another possibility is that the somewhat gradual onset of the mechanical stimulation gave rise to SEPs skewed in latency or multiple, overlapping early SEPs. Given the zero-bound constraint in random effects, a skewed distribution would be expected. Early ERPs would therefore be predicted to have a delayed and temporally blurred onset, as can indeed be observed from the smaller amplitude and wider area of the early mechanical potentials displayed in Fig. 4. As this invalidates peak amplitudes^18^, we omitted the mechanical stimuli from the analysis of early effects.

### Source localization of early SEPs

We first set out to validate that the early potentials were indeed related to primary somatosensory processing of tactile stimuli, as opposed to higher order processes. For this to be the case, they should show stronger activation in somatosensory-related areas at higher intensity input levels. Secondly, we used source localization of activity to inform whether the three potentials could be dissociated on the basis of their neuronal generators.

The exact low resolution brain electromagnetic tomography (eLORETA) technique, as implemented in LORETA-KEY software was used to compute three-dimensional source localized activity over each participant’s low- and high-intensity averaged vibration-evoked potential. The eLORETA method is a recent, discrete, linear, weighted minimum norm inverse solution which provides current density images of source localized activity, albeit at a low (i.e. blurred) resolution. It is similar to the widely used LORETA^19^ and standardized LORETA^20^ methods, but arguably improved as it has no localization bias even in the presence of structured noise^21^.

Following projection of the evoked potentials onto 2394 voxels of the cortical grey matter and hippocampus of the solution space, the activity was averaged within the windows defined for the three components. We then tested the difference between the high and low intensity images, correcting the critical thresholds and p-values by performing randomization statistical non-parametric mapping. This showed, for the P25, the peak difference to be in the left Brodmann Area 5 (BA5), at MNI coordinates [−20, −45, 50], extending to BA3. The P30 was localized more anterior, with peak difference in the left BA6 at [−35, −5, 60], also extending to the left BA7, BA4 and BA5. The N50 showed similar activation to the P25, with peak difference at [−20, −45, 50], but with a wider area of activation, including the left BA7, BA6, BA40 and BA5. In general, this suggests all three potentials are localized broadly in sensory-motor areas, although the P25/N50 can be more readily related to tactile spatial functions^22^,^23^, while the more frontal areas of the P30 may be associated with tactile object recognition^22^. However, it should be noted that the source localization here was based on only 32 channels of EEG without digitization of the locations of electrode positions, resulting in far lower accuracy than can be expected with other imaging solutions.

### Effects of emotional expression on early SEPs

To test the effect of expressed emotion on early potentials, three independent three-factor repeated-measures ANOVAs with *emotional expression* (neutral, anger, happiness, fear, sadness), *touch intensity* (soft, hard), and *electrode* (CP5, C3, FC1, FC2, C4, CP6) were conducted. In order to reduce the likelihood of type I errors, given the three separate analyses, we used an alpha of p = 0.0167 (0.05/3).

For the P30, *electrode*, F (1.80, 70.29) = 17.80, MSE = 8.04, η^2^ = 0.31, p < 0.0001, and *touch intensity*, F (1, 39) = 7.74, MSE = 1.46, η^2^ = 0.17, p = 0.008, were significant main effects, while *emotional expression*, p > 0.3, was not significant. The *electrode* effect showed lateralization of the P30, with positive amplitudes over the left hemisphere (strongest over C3) and negative amplitudes over the right. The P30 became (0.12 ± 0.05 μV) more positive with higher touch intensities. No significant interactions were found, *p*s > 0.15.

For the P25, *electrode*, F (2.30, 89.76) = 27.91, MSE = 3.28, η^2^ = 0.42, p < 0.0001, and *touch intensity*, F (1, 39) = 6.73, MSE = 1.42, η^2^ = 0.15, p = 0.014 were likewise significant. Here, the topography had maximal positivity over FC1 (0.48 ± 0.08 μV) and FC2 (0.37 ± 0.07 μV) and negativity over CP5 (−0.27 ± 0.06 μV) and CP6 (−0.30 ± 0.05 μV). The P25 increased (0.13 ± 0.05 μV) with *intensity*. The interaction between *electrode* and *intensity* was also significant, F (2.32, 90.60) = 4.36, p = 0.012. In order to probe this effect, we then conducted the same ANOVA separately for each channel. The results revealed that touch intensity was positively related to P25 activity, but only in contralateral FC1 (p = 0.005) and C3 (p = 0.001) sites. *Emotional expression* had neither a main-effect, nor entered in a two-way interaction effect, *p*s > 0.1. However, there was a significant three-way interaction, F (8.28, 323.08) = 2.78, MSE = 1.47, η^2^ = 0.07, p = 0.005. As shown in Fig. 5 (left panel), there was a more pronounced lateralized *touch intensity* effect after anger and happiness than after other emotions. To better understand the effect, we then computed sixteen post-hoc comparisons in which average differences between the four emotional expression conditions and the neutral condition were computed independently over the left and right hemisphere for the soft and hard *intensity* conditions. This revealed that larger P25s were observed in the left hemisphere with strong tactile stimuli following anger (p = 0.0003) or happiness (p = 0.0005).

The effects on N50 were similar to those of P25, with significant effects of *electrode*, F (2.26, 87.96) = 96.22, MSE = 12.27, η^2^ = 0.71, p < 0.0001, and *touch intensity*, F (1, 39) = 12.27, MSE = 1.26, η^2^ = 0.24, p = 0.001, but not *emotion*, p > 0.6. The interaction between *electrode* and *touch intensity* was again significant, F (2.68, 104.51) = 14.55, MSE = 1.88, η^2^ = 0.27, p < 0.0001, as was the three-way interaction, F (6.94, 269.71) = 2.62, MSE = 2.30, η^2^ = 0.06, p = 0.013. Figure 5 (right panel) shows that strong touch preceded by happy, angry, and sad expressions elicited pronounced contralateral negativity. To confirm this observation we then conducted two follow-up ANOVAs separately for soft and hard touch where expression and intensity were included as factors and N50 laterality as a dependent variable*. A significant interaction between expression and intensity was observed, but only for strong touch intensities (soft touch: p = 0.59, hard touch: p = 0.004). Within the strong touch condition, pairwise comparisons of facial expressions revealed stronger N50 in response to sad vs. neutral (p = 0.038) and angry vs. fearful (p = 0.049, Bonferroni adjusted) expression.

### Late SEPs

The effect of *emotional expression* (neutral, anger, happiness, fear, sadness) on late potentials was first tested with a single five-factor repeated-measures ANOVA with *electrode* (CP1, CP2, Cz, P3, Pz, P4), *touch type* (vibration, mechanical), *touch intensity* (soft, hard), and *time* (100–200, 200–300, 300–400, 400–500, 500–600, 600–700) as factors. *Electrode*, however, did not significantly interact with the variables of interest and was removed from the model for the sake of parsimony (but see supplementary material for the full factorial results). Significant main effects of *time*, F (1.94, 75.56) = 11.89, η^2^ = 0.23, MSE = 100.40, p = 0.00004, and *emotional expression*, F (3.77, 147.12) = 6.19, η^2^ = 0.14, MSE = 22.36, p = 0.0002, but not of *touch type*, p = 0.58, or *touch intensity*, p = 0.33, were observed. As shown in Fig. 6, amplitudes increased between the first (100–200) and third (300–400) bin before slowly declining.

More importantly, the effect of *emotional expression* showed highest late SEP amplitudes after anger, lowest after happiness, with fear, neutral and sadness in between (see Fig. 6). *Bonferroni* adjusted post-hoc comparisons showed that this effect was mainly driven by the difference between happy and angry touch, t (39) = 4.61, p = 0.0004, although neutral, sad, and fearful touch all evoked higher amplitudes than happy touch as well, *p*s < 0.05. *Emotional expression* furthermore significantly interacted with *time*, F (8.71, 339.71) = 3.82, η^2^ = 0.09, MSE = 3.37, p = 0.0002, and with *time-*by-*touch intensity*, F (9.71, 378.49) = 2.36, η^2^ = 0.06, MSE = 3.10, p = 0.01. To understand the temporal localization of the effect of *emotional expression*, we performed independent ANOVAs for the six different bins, averaged across *touch types* and *touch intensities*. First, this showed significant effects of *emotional expression* for all bins, except the first (successively p = 0.69, 0.00803, 0.00063, 0.00010, 0.00015, 0.00003). Second, time courses of the anger and happiness effects differed: as shown in Fig. 6, the amplified effect of anger was already significantly higher than neutral at 200–300 ms (p = 0.006), while happiness was significantly lower than neutral at 400–500 ms (p = 0.01).

*Touch type* and *touch intensity* did affect the somatosensory potential but no other interactions with *emotional expression* were observed (see table in Supplementary Material).

### Trait-level associations

Given the converging effects of *emotional expression* on the late SEP and expression ratings, we speculated that the late SEPs were related to the more affective, interpersonal aspects of the touch. To explore this possibility, we calculated four averages of *avatar ratings*, one for each subjective judgement, and averaged across *emotional expressions*. Following that, we compared these with the late SEP, likewise averaged over conditions. This showed positive correlations between SEPs and *avatar pleasantness*, R = 0.41, p = 0.007, but not between SEPs and *touch pleasantness*, R = 0.23, p = 0.09, or either *intensity* rating, *p*s > 0.6.

## Discussion

People process touch differently depending on the surrounding socio-emotional cues^6^. This contextual modulation has been shown to take place in the somatosensory and other cortical structures^13^,^24^. So far, however, it has been unclear whether the modulation takes place also in the context of interpersonal touch, and if so, at what level of processing the modulation actually occurs^5^. To increase our understanding of the affective modulation and its temporal nature, we investigated how a toucher’s emotional expressions modulate ERP activity elicited by interpersonal touch.

Along the lines of earlier findings, our results supported the notion of affectively modulated touch perception. When explicitly asked about a touch, the participants rated the same touch as more intense when preceded by an angry (clearly most intense), happy or fearful expression than when a sad or neutral expression was shown. This was paralleled by the findings on *early* SEPs, with amplified, left-lateralized P25 and N50 activity after angry and happy expressions. In the range of N50, however, a negative peak was also amplified after a sad expression. Altogether, the findings suggest that the affective modulation initiates already during subcortical processing. In earlier research, similar findings have been obtained when investigating attentional amplification of visual and auditory sensory processes^25^,^26^. In these studies, the attended stimuli have been shown to elicit modified processing in the thalamus.

The present findings suggest that similar top-down influences can result from affective relevance of the stimulus. Moreover, the differences between expressions suggest that motivational anticipation rather than emotional valence might underlie the early modulation. Given that both early SEPs responded selectively to the angry- and happy-expression touch and these two emotions, contrary to the others, share the same approach-motivation^27^, it can be assumed that the early processing is particularly sensitive to toucher’s motivational state. However, while an approach tendency has been associated with left-lateralized frontal activity^28^, here we find an effect that seems more like an increased tactile sensitivity of the hemisphere contralateral to the touch. This is more in line with the tactile affordances that can be attributed to the emotional states: after some expressions touch follows more naturally and is thus attentionally amplified in early processing. This interpretation is also supported by the observations that N50 was also amplified after sad-expression touch. We will further describe the early effects as being of an anticipatory nature below.

The late effects were likewise strongest after the happy and angry expressions. This was, however, the only similarity with the early effects, as the topography, specificity, and direction of the effects were all markedly different for the late effects. First, the P200 responses were not lateralized, despite the touch occurring only on the right hand. Second, although the physical parameters of the touch affected the amplitude of the P200, these effects were additive to the effects of emotional expression and did not, as opposed to the early effects, interact. Third, although the touch after an angry expression was again found to amplify the potentials, happy expressions resulted in suppressed P200s. Interestingly, this pattern of results paralleled self-reported pleasantness of the avatar and touch.

Given the differences in the latency, topography, specificity, and directionality of effects, the results strongly indicate a dissociation between two stages of emotional touch perception. The first, “anticipatory” stage is putatively pre-attentive and pre-conscious, as indicated by the latency of the effects. The specificity and latency of its effects indicate these emotional modulations operate in a top-down manner on tactile perception, as it is unlikely that any cortical processing of somatosensory activity has taken place at this stage. The second, “conscious” stage is late and general, which suggests that it takes place after cortical somatosensory processing – possibly after the initial activity is recognized as representing interpersonal touch. It could thus be characterized as a bottom-up type of modulation. Together, the two stages may account for the previous, divergent results related to the emotional effects on early and late tactile perception.

### Stage 1: Emotion and touch anticipation

We argue that the first stage concerns top-down affective modulation of subcortical-cortical pathways. The latency itself poses serious constraints on the possible neural structures involved: given that the somatosensory cortex itself is only activated 20 ms after median nerve stimulation^29^, it is unlikely that recurrent activation from regions beyond the SCx are involved. This suggests that the affective modulation concerns the pathway between SCx and the immediately preceding areas, such as the thalamus. The notion that the thalamus is involved in emotions goes back to the early 1900s^30^,^31^, and recent neuroimaging research showed, for example, that both positively and negatively valenced emotional pictures resulted in increased regional blood flow in the thalamus^32^. One could, therefore, speculate that the emotional modulation operates on the thalamus, explaining how both the early effects of SEPs as well as subjective judgments of tactile intensity are enhanced after perceiving anger and happiness.

It is possible, however, that the early modulation is not related only to the approach-withdrawal continuum since sad-expression touch also amplified the early SEPs. Alternatively, the early modulation may reflect the top-down influence of a person’s expectations. Indeed, in some earlier ERP studies, participants’ attention and expectations have been shown to modify early SEPs^33^,^34^. Interestingly, this modulation also takes place as early as 50 ms post stimulus^34^. In a natural face-to-face context people may use facial expressions in order to prepare the receiver to tactile communication. Perhaps neutral or fearful expressions are less clearly conveying other’s motives to reach out and touch, whereas, in a hostile, affiliative, or sad mood, people may seek physical contact. Thus, we suggest that the early phase of somatosensory processing is primarily affected by a perceiver’s expectations of others’ further actions.

### Stage 2: Emotion and touch interpretation

The second stage sets in well after the SCx is activated and could therefore involve feedback processes that rely on the tactile stimulation being detected as a significant type of signal. Functional MRI studies have previously implicated a wide network of cortical areas as being related to the affective dimension of touch, such as the posterior insula, parietal cortex BA 7, anterior cingulate cortex, ventral striatum and orbitofrontal cortex^35^,^36^,^37^. Functional MRI lacks the temporal resolution of EEG, but in the light of the present study, we assume these structures are related to the second stage: the later activity was found to reflect both the emotional meaning of the touch and its physical, tactile parameters, but not the interaction between the two. Accordingly, the stage follows initial identification or anticipation of the tactile sensation as an interpersonal touch, which is subsequently processed in similar ways to other tokens of its type.

In that sense, the somatosensory P200 seems related to the more familiar P300 in visual and auditory evoked potentials, which is commonly associated with post-attentive processes, such as updating memory^38^, response preparation^39^, and consciousness^40^. This may seem early for a P3 component to occur, but somatosensory potentials often precede their counterparts in other modalities. For example, the tactile P30 in the present study could map onto the visual C1 in the primary visual cortex^41^ with a peak latency of ca. 60 ms later. Later processing does necessarily remove this precedence effect: for example, a component for tactile spatial attention was recently observed at 140 ms, ca. 110–150 ms earlier than the functionally similar visual N2PC^42^.

Finally, the effects on P200 appear to mirror the subjective interpretation of touch. Angry expressions elicited more negative affective ratings of touch and increased P200s in response to the touch, while happy expressions were associated with more positive touch ratings and suppressed P200s. It appears then, that at this stage, participants extract meaning from the tactile feedback. Perceiving the emotional expression of others is well-known to affect one’s own emotion^43^,^44^, which we suggested is used as a source of information to interpret touch. This explains why the affective rating of touch closely followed that of the emotional expression: what we perceive on our skin is partially determined by how we feel.

Interestingly, while within a participant, P200s were associated with angry expressions and more negative affective ratings, between participants, the P200 correlated positively with perceived avatar pleasantness (irrespective of emotional expression). Thus, it appears that a dispositional tendency to perceive others as pleasant may be rather strongly associated with a propensity to react with large P200s to touch. While the reason for this finding remains unclear, it seems to suggest that encoding the social meaning of touch is also affected by a perceiver’s social characteristics.

Of course, one may argue that the stimuli used in the present study were rather artificial, and that vibrations and mechanical sensations are unlike the feeling of the warm skin from another human being. Indeed, some scholars consider only a slow caress as an affective touch^37^. There is also evidence that a touch delivered using the experimenter’s hand elicit larger responses than a touch delivered through a velvet stick in the contralateral primary and secondary somatosensory areas and posterior insula^45^. However, in some other studies, human-delivered and mechanical touch have not been found to differ in terms of affective outcomes^7^,^12^,^46^. It is also of note that the use of mediated touch, instead of a real physical contact, can also be considered as an important methodological strength of the present study since it enhanced the controllability and consistency of the touch which would not be possible in real skin contact.

In summary, the present study suggests that at the first, anticipatory stage, our somatosensory processing is particularly sensitive to the facial expressions that better prepare for the tactile communication, whereas at the second stage, tactile processing is mainly guided by the valence of a toucher’s expression. We were able to show that people attribute a remarkable degree of human emotion to perceive interpersonal touch, although the touch was always the same vibrotactile or mechanical simulation. The earliness of affective modulation suggests that the emotional context in which a touch is delivered is an inseparable aspect of interpersonal physical contact.

## Methods

### Participants

Forty-three Finnish university undergraduates volunteered to take part in the study in exchange for a monetary compensation. Due to technical problems, data from three were removed from the analysis. Of the remaining, seventeen participants were female and twenty-three male. They were right-handed, had no history of neurological or mental disorders and were between 25.0 ± 4.0 years of age. Participants were informed of their rights, including their right to withdraw from the study at any moment without fear of negative consequences, and of the content and purpose of the experiment before signing informed consent. The study conformed to the guidelines laid out by the declaration of Helsinki and was approved by the Aalto Ethics Research Committee.

### Stimuli and Apparatus

The experimental paradigm was programmed using the Unity 3D 4.5.4 platform (Unity Technologies, San Francisco, CA). The experiment was run on a PC running Windows 7, synchronizing stimulus presentation and behavioral data recording with additional apparatus: a tactile stimulus generation mini-computer, a head-mounted display (HMD) and a hand-tracking device.

### Emotional expressions

A professional actor was recruited prior to the experiment in order to obtain dynamic expression data. Emotional expressions were recorded with Faceshift software (Faceshift AG, Zürich, Switzerland), which utilized a Microsoft Kinect depth camera to quantify facial shape markers in time and space. Parameters thus obtained were projected onto a single male avatar, which was created using the default head model of Faceshift software and body designed with Fuse modelling tool provided by the Mixamo platform (Adobe Systems, San Jose, CA). Expression projections were used to create five unique 4 s animations (with neutral onset expression) for 6 emotional (anger, fear, happiness, surprise, disgust and sadness) and one neutral (control) expressions. The initial set of 35 animations was pre-tested with an independent group of 14 participants, showing reasonable recognition performance overall (73.3%), but low performance for disgust (55.4%) and surprise (62.5%). To improve the accuracy for the present experiment, we omitted these emotions and used only the three animations of the other expression with the highest recognition performance.

### Tactile stimuli

A glove was custom designed to standardize the placement and pressure of tactile actuators across participants. Tactile stimulus was always presented on the right hand and was delivered by one of two tactile technologies. Vibrotactile stimuli were presented using TEAX14 C02-8 audio-exciters (Tectonic Elements ltd., St. Neots, United Kingdom, www.tectonicelements.com), placed on the dorsal of the right hand, to the middle of the metacarpal bones, and posterior to the little finger and thumb. Mechanical stimuli were presented using a OmG 9 g micro servo motor (maximum speed 60°/0.1 s, torque 1.3 kg/cm, RC OMG, Shenzhen, China) stretching ca. 10 cm of elastic tape over the volar, producing mild tension mainly at sites lateral, and 1 cm posterior to, the first major knuckles of the index and little finger. Both stimulus types had a duration of 0.5 s. Vibrations were 35 (soft) or 100 Hz (hard) square wave sinusoids of 0.5 s duration and constant amplitude. Mechanic stimuli were varied in intensity using the rotation of the motor between 120° (soft) and 180° (hard). Timing of tactile stimuli was optimised using an Arduino Uno R3 micro controller (Arduino Inc., Ivrea, Italy). The onset of tactile stimuli was measured independently with accelerometers (Brain Products GmbH, Gilching, Germany) positioned within 1 cm from the actuators. Both touch types produced extraneous sound, for which reason participants wore ear plugs. Furthermore, a masking sound was played throughout the experiment. This was created by recording and sampling the sound of the various touch types and intensities as a looped pattern mixed with white noise.

### Interaction in virtual reality

A head-mounted display (HMD) enabled the virtual 3D environment. The Oculus Rift DK2 (Oculus VR Inc., San Francisco, CA) was used, running at a resolution of 960 × 1080 px per eye and a refresh rate of 60 Hz. It allowed 3D virtual reality at a field of view of 100° on average by combining motion parallax and stereoscopic cues. Virtual reality in the experiment was enhanced by measuring the head movement at 1000 Hz using the HMD’s integrated 3-axis accelerometers, gyroscope and magnetometer and using the information to displace the camera position in the virtual environment.

**Figure 2:**
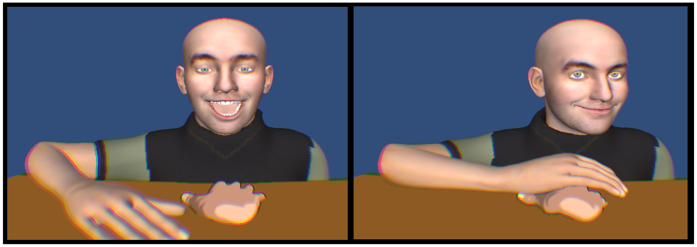
Experiment paradigm. A 3D modelled virtual person expressed an emotion before reaching out to the participant’s virtual hand. A tactile signal (see Fig. 1) was presented when the hands touched.

**Figure 3:**
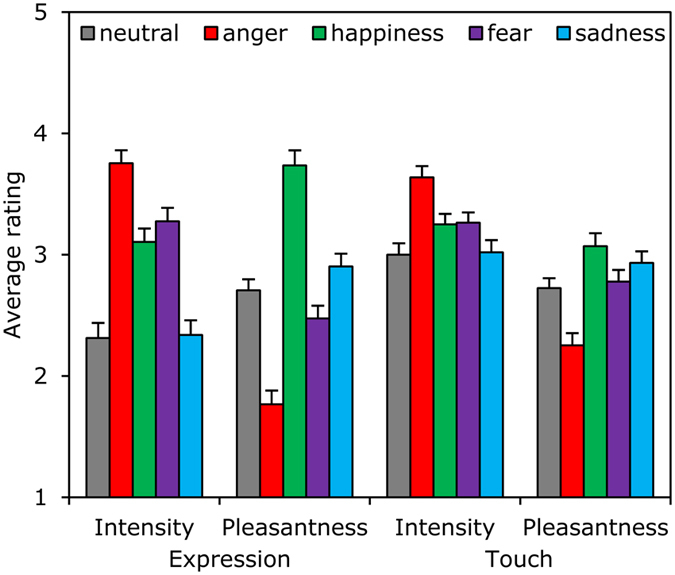
Emotions affect self report. Average ratings of intensity and pleasantness of avatar and touch are displayed as a function of preceding emotion. Error bars indicate standard errors of mean.

A hand-tracking device enabled agency over a virtual version of participant’s hand. The Leap Motion (Leap Motion Inc., San Francisco, CA) controller was positioned 16 cm below a glass table on which participants held their right hand. This provided data concerning the hand and joints positions that were used to project a dynamically updated (at 60 Hz) visual representation of the hand within virtual reality (see Fig. 2).

### Procedure

Following instruction and informed consent, participants were seated at a desk and asked to place their hand on the glass table. They were then provided assistance in putting on the HMD and fitting the tactile glove. Within virtual reality, they were shown the general stimulus scenario including a green area proximal and right of the centre of a brown table, as well as their virtual hand. Moving the hand over the green area caused the area to disappear and a blue crosshair to appear in the centre instead. Simultaneously, the avatar was shown, positioned at a virtual distance of 0.76 m. Its face had a width of ca. 12.8° and was initially presented wearing a neutral expression. Moving the hand to the blue crosshair started the emotional expression animation. After a randomized interval of 1–3 s, the avatar moved his right hand toward the participant’s hand. This point was reached after a constant 1 s, at which point the tactile stimulus was presented (i.e. 2–4 s after expression onset). After the tactile duration, and a further 0.5 s, either the next trial was started or questionnaires were presented.

Questionnaires concerned the touch experience, emotional experience, or the emotion recognition. To avoid confusion regarding the target of the questionnaires, we separated the questionnaires between blocks. That is, in the first 20 trials of each block, trials ended with questionnaires. In the first blocks, items concerned the expression’s pleasantness, intensity, humanity, forcefulness and friendliness (e.g. “Was the emotional expression friendly?”), with participants using their left hand and the arrow keys to indicate their agreement on continuous Likert scales (1: “not at all friendly”, 5: “very friendly”). In the second block, participants were shown the same items but now concerning the tactile sensation (e.g. “Was the touch intense?”). The last three blocks included the five-alternative forced-choice emotion recognition task, in which they were instructed to select the adjective that best described the avatar’s emotion (angry, happy, sad, afraid or neutral).

Every block had 100 trials, with the order of each combination of 2 touch types (vibration or mechanical), 2 intensity levels (soft or hard), 5 emotions (anger, fear, sad, happy or neutral) randomized within every 20 trials. The entire experiment consisted of 5 blocks, with self-timed breaks provided in between, lasting around 90–120 minutes in total excluding EEG preparation.

### EEG Recording and Pre-Processing

EEG was recorded at 1000 Hz from 32 Ag/AgCl electrodes using a QuickAmp (BrainProducts GmbH, Gilching, Germany) amplifier. The electrodes were positioned on equidistant electrode sites over FP1, FP2, F7, F3, Fz, F4, F8, FT9, FC5, FC1, FC2, FT10, T7, C3, Cz, C4, T8, TP9, CP5, CP1, CP2, CP6, T8, P7, P3, Pz, P4, P8, O1, Oz and O2 with AFz as ground using EasyCap elastic hats (EasyCap GmbH, Herrschin, Germany) and referenced to the common average. Horizontal electro-oculographic (EOG) activity was recorded with two electrodes placed ca. 1 cm lateral to the outer canthi of each eye, and vertical EOG was recorded from electrodes ca. 1 cm inferior and superior to the left eye. EEG and EOG were pre-processed off-line with 0.1 Hz high-pass and 50 Hz notch filters. Artifact correction was based on independent component analysis (ICA) using the logistic infomax algorithm as implemented in EEGLAB^46^. The data were first filtered with an extra low-pass filter at 80 Hz, and segmented to include 1 s following the touch and 7 s before the touch. Segments with extreme (>500 μV) were removed (ca. 4.4%) to not bias ICA toward extremities. The data then entered ICA after the activity, frequency spectrum and topography of components were visually inspected for the presence of eye-movement, eye-blink and other artifacts. The EEG was then reconstructed by using these weights for the unfiltered, continuous data and projecting only artifact-free components to the electrode level.

Note that it is possible that use of the HMD caused a degree of noise. However, interference caused by the HMD should be independent from EEG, for which reason ICA can remove the interference without necessarily affecting signal, as has previously been shown^47^. To find out how strongly the HMD affected the EEG, we compared the ICA from the present experiment with the same participants’ data collected in a different study (which they undertook randomly before or after the present one). The same subjects had 3.1 ± 0.6 more components (of 34) marked as artifactual in the HMD experiment, suggesting an upward limit of additional independent interference related to use of the screen.

### ERP measurements

Analysis of event-related potentials was carried out in Brain Vision Analyzer (BrainProducts GmbH, Gilching, Germany) and included segmentation into 1 s epochs, including 200 ms of EEG prior to the tactile stimulus and 800 ms of event-related activity. Segments were removed using thresholds at 40 μV absolute amplitude and 60 μV peak difference. This removed ca. 18.7 ± 12.3% of segments, with 20.3 ± 3.1 trials for each of the 20 analysis cells. For peak analysis of early potentials, a low-pass filter of 40 Hz was used. No additional filtering was applied for the analysis over late potentials.

**Figure 4:**
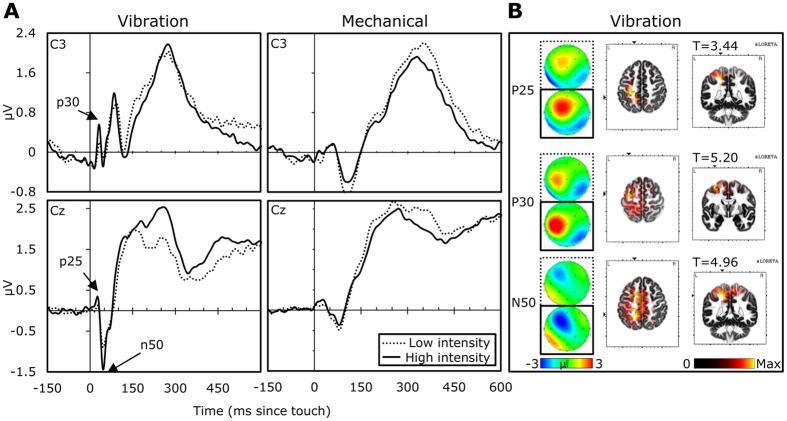
Somatosensory potentials evoked by vibrations and mechanical stimulation. A: temporal localization of P25 and N50 (in Cz) and P30 (in C3), and effects of high (straight lines) and low (dotted lines) intensity. B: Effect of intensity on scalp topography and source localized activity. Topography shows current source density in high (straight lines) and low (dotted lines) conditions with the scalp map displaying activity from all recorded channels. Source localized activity shows the significance of the eLORETA backprojected difference between them.

In order to identify early SEPs, we first computed and visually inspected the grand average ERPs of the hard mechanical and vibrotactile stimuli, irrespective of preceding emotion. These showed strongest amplitudes in central-left channels for early SEPs (see Fig. 4). Following that, we used a peak detection algorithm based on the first 60 ms of grand average activity divided by its standard error with a threshold of T(39) = 4. For vibrotactile stimuli, an early potential was observed in Cz (22–29 ms, max T(39) = 4.88 at 26 ms), followed by negativity (36–66 ms, max T(39) = 10.86 at 47 ms). We named these P25 and N50 respectively. C3 showed a different pattern, with a peak value in between P25 and N50 (24–41, max T(39) = 7.72 at 31 ms). This potential was found to have a markedly different topography to the P25 (see Fig. 4), for which reason we analysed it independently and will refer to it as the P30.

**Figure 5:**
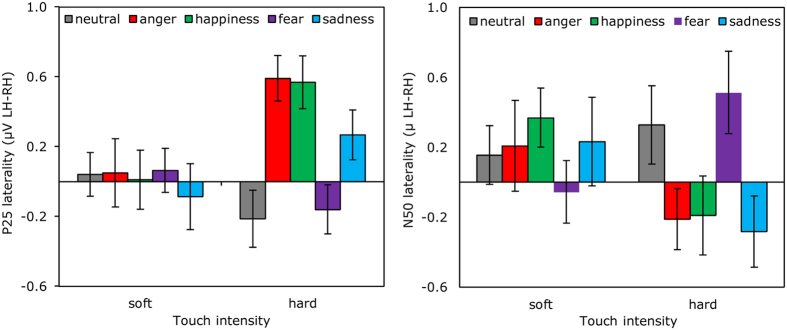
Emotions affect early somatosensory potentials. Laterality of the early somatosensory potentials (P25 on the left and N50 on the right) is displayed as a function of preceding emotion and stimulus intensity. Laterality index was computed distracting averaged left hemisphere ERP activity from the corresponding right hemisphere activity*. Error bars indicate standard errors of mean.

**Figure 6:**
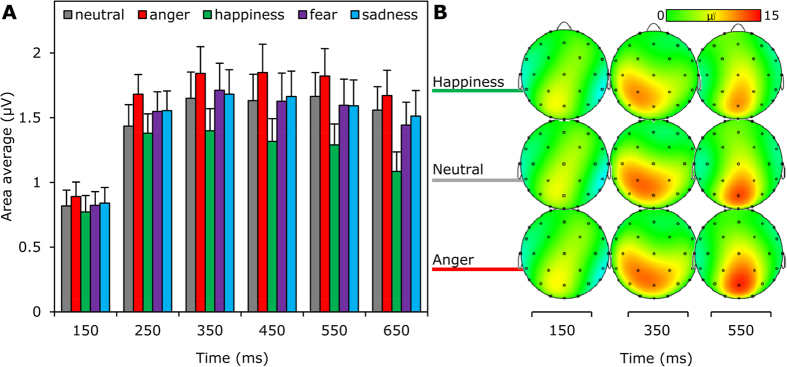
Emotions affect late somatosensory potentials. A: Average voltage of the somatosensory potential for six 100 ms bins following the first 100 ms post stimulus onset. The bars display bins of 100 ms and are averaged across centro-parietal areas (Cz, CP1, CP2, P3, Pz, P4) centered around the midpoint indicated on the horizontal axis with error bars indicating standard errors of mean. B: The topography of three bins is provided to show how anger and happiness are dissociated from neutral conditions.

Following, latencies of local peaks were extracted for each condition and P25, N50 and P30 time-ranges, after which the analysis centred on the voltage across sites overlying sensorimotor (CP5, C3, C4, CP6) and central (FC1, FC2) areas of both hemispheres. For the very first occurring SEPs, i.e. the P25 and P30, the peak (local maximum) amplitude was used, while for N50, the peak-to-peak difference with P25 was used to avoid confounding the effects of P25 and N50. Visual inspection of the grand averages showed a later, longer-lasting component similar to the somatosensory P200^14^ and P300^12^, with the latter previously having been shown to be modulated by affective relevance. Rather than defining a plurality of areas and increasing the chance of type-I errors, we used 6 bins spanning the breadth of the P200 and P300 as well as a potential later positive component previously suggested to capture motivational significance^28^. Within this area, we observed high amplitudes particularly over the centro-parietal areas (see Fig. 5), for which reason we used amplitude averages over Cz, CP1, CP2, P3, Pz and P4.

### Design

The experiment used a within subject design with *emotional expression* (neutral, angry, happy, fear, sad), *touch type* (vibration, mechanical) and *touch intensity* (soft, hard) as factors. For behavioural data, four ANOVAs were conducted with pleasantness and intensity of touch and expression as dependent variables. For early SEPs, the analysis additionally included *electrode* as factor (CP5, C3, FC1, FC2, C4, CP6) and three four-way repeated-measures ANOVAs were conducted with peak amplitude of P25, P30 and N50 as measures. For late SEPs, a single repeated measures ANOVA with average amplitude as dependent and was conducted with additional factors of *electrode* (CP1, CP2, Cz, P3, Pz, P4) and *time* (100–200, 200–300, 300–400, 400–500, 500–600, 600–700 ms). The Greenhouse–Geisser correction was used when necessary to correct for non-sphericity.

## Additional Information

**How to cite this article**: Ravaja, N. *et al*. Feeling Touched: Emotional Modulation of Somatosensory Potentials to Interpersonal Touch. *Sci. Rep.* **7**, 40504; doi: 10.1038/srep40504 (2017).

**Publisher's note:** Springer Nature remains neutral with regard to jurisdictional claims in published maps and institutional affiliations.

## Supplementary Material

Supplementary Information

## Figures and Tables

**Figure 1:**
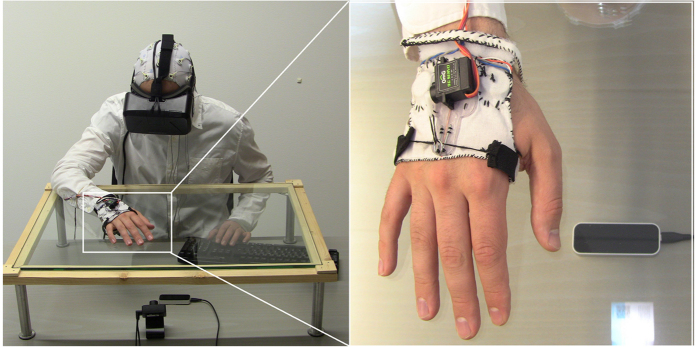
Experiment setup. Head-mounted displays were worn over EEG caps to enable virtual reality. A hand-tracking device was positioned underneath a glass table and enabled sight of hand in virtual reality. The tactile glove enabled vibrotactile (actuators near the thumb) and mechanical stimuli (motor pulled wires, stretching fabric near knuckles).
