# Supplementary material for: Feeling Touched: Emotional Modulation of Somatosensory Potentials to Interpersonal Touch
Source: Sci Rep. 2017 Jan 12;7:40504. doi: 10.1038/srep40504 (PMC5228183; doi:10.1038/srep40504)
Supplement: Supplementary Information [file srep40504-s1.pdf]

Feeling Touched: Emotional Modulation of Somatosensory Potentials to Interpersonal Touch

Ravaja, N., Harjunen, V., Ahmed, I., Jacucci, G., & Spapé, M.M.

Supplementary information 1

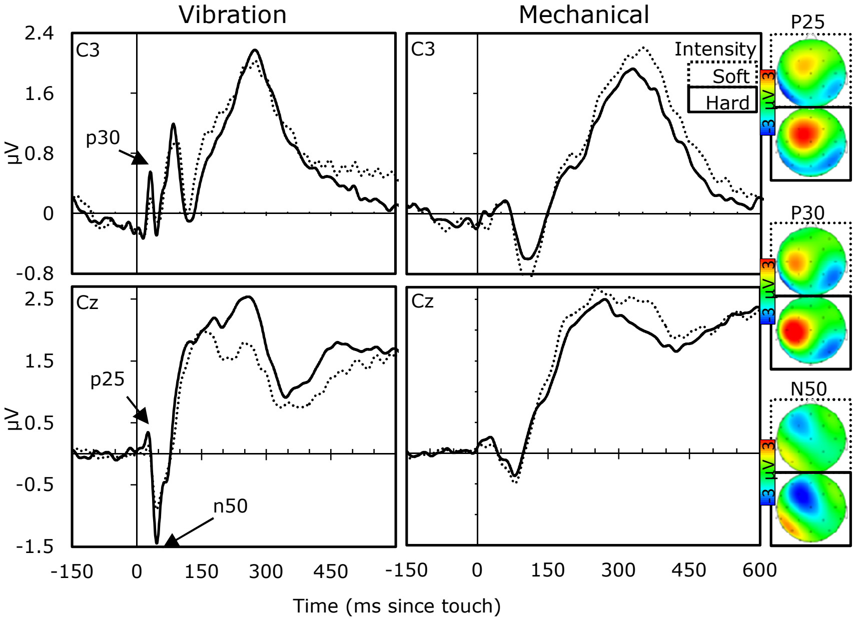

Supplementary information 2

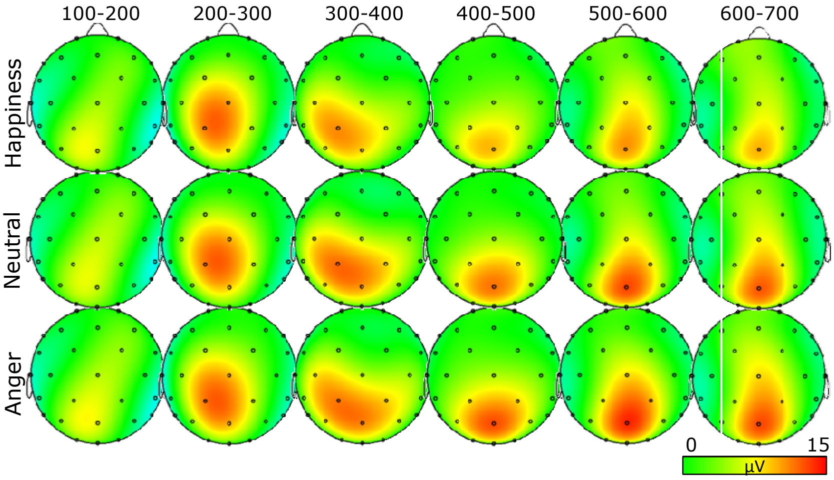

Supplementary information 3

Table 1:
Late effects of touch

Emot.	Time	Touch	Intens.	Elec.	df	F	η²	p
					4,147	6.19	.14	*****
					2,76	11.89	.23	*****
					1,39	0.31	.01	
					1,39	0.97	.02	
					3,100	25.88	.40	*****
					9,340	3.82	.09	*****
					4,140	1.53	.04	
					3,133	28.66	.42	*****
					4,152	1.97	.05	
					2,97	4.93	.11	**
					1,39	1.99	.05	
					10,376	0.79	.02	
					6,220	23.76	.38	*****
					2,81	11.97	.23	*****
					4,139	2.55	.06	*
					9,363	1.61	.04	
					10,378	2.36	.06	*
					4,140	0.80	.02	
					3,116	5.19	.12	***
					18,695	1.70	.04	*
					11,421	1.12	.03	
					6,250	19.62	.33	*****
					11,434	0.85	.02	
					9,338	3.60	.08	*****
					3,130	2.85	.07	*
					9,340	0.77	.02	
					19,737	1.02	.03	
					18,696	0.94	.02	
					11,420	1.25	.03	
					7,270	7.10	.15	*****
					19,732	1.11	.03	

Note. White shading indicates presence of factors in test, significance abbreviated as *: < .05; **: < .01; ***: < .005; ****: < .001; *****: < .0005; The presence of factors is with white shading (e.g. the sixth row shows the interaction between time and emotion to be significant at p < .0005).
